# Clinico-pathological features of bladder carcinoma in women in Pakistan and smokeless tobacco as a possible risk factor

**DOI:** 10.1186/1477-7819-3-53

**Published:** 2005-08-05

**Authors:** Muhammad Rafique

**Affiliations:** 1 Department of Urology, Nishtar Medical College, Multan, Pakistan

## Abstract

**Background:**

Bladder carcinoma is one of the common urological malignancies occurring worldwide in both sexes. Use of smokeless tobacco by women is common in rural areas of Pakistan. The clinico-pathological features of bladder carcinoma in women and association of smokeless tobacco as a possible risk factor for bladder carcinoma has not been well described in the literature. The objective of the study was to determine the clinico-pathological features of histologically confirmed bladder carcinoma in women and to investigate the role of smokeless tobacco use as a possible risk factor for its development.

**Patients and methods:**

Of the 204 patients (160 male and 44 female M:F ratio 3.6:1) of newly diagnosed bladder carcinoma treated at Nishtar Medical College Hospital Multan from January 1998 to December 2004, the 44 female patients were evaluated with respect to age, clinical presentation, cystoscopic findings, histopathological reports and possible etiological factors. Data were collected and prospectively updated at the time of discharge from hospital and during follow-up in urology out-patient clinic.

**Results:**

Transitional cell carcinoma accounted for all of the bladder carcinoma in women. Median age of the patients was 55 years and 68% patients were under 60 years of age. Majority of patients (88%) presented with hematuria. Eleven (25%) patients had superficial (pTa/pT1) while 33 (75%) patients had muscle invasive (T2–T4) bladder carcinoma. Most (81%) superficial tumors were papillary while muscle invasive tumors had solid configuration at cystoscopy. Of these, 21 (47%) patients had long history of smokeless tobacco use (chewable or moist snuff).

**Conclusion:**

Transitional cell carcinoma is the most common bladder malignancy in women in Pakistan. Many women with bladder carcinoma had long history of use of smokeless tobacco. Majority of patients presented with hematuria and were under 60 years of age. At the time of diagnosis 75% women had muscle invasive bladder carcinoma. In women using smokeless tobacco, the correlation between stage of bladder carcinoma and duration of smokeless tobacco use was significant (*p *= *0.03*). Further studies are needed to clarify the role of smokeless tobacco in the development of bladder carcinoma.

## Background

Bladder carcinoma is one of the most common malignancies occurring worldwide. It is seen mainly in men. The incidence in women is approximately 3 to 4 times lower than in men but it seems to be rising [1]. Bladder cancer has been associated pathogenetically with many etiological factors which include occupational exposure to certain chemicals e.g. aniline dyes, cigarette smoking, viral agents, bacterial and parasitic infections, cystolithiasis, cyclophosphamide therapy and pelvic irradiation [2].

The initial clinical evaluation consists of history and physical examination, upper tract studies (IVU +/- Ultrasonography) and urine cytology followed by cystoscopy and transurethral resection of bladder tumor.

Most cases of the bladder carcinoma are superficial at the time of diagnosis (stage Ta-T1). The recurrence of the superficial tumors can be as high as 70%, with 10–15% progressing to muscle invasive disease [3].

Despite the fact that bladder carcinoma is among one of the common malignancies in women worldwide, the etiological and clinico-pathological aspects of bladder carcinoma are not well described in the literature. In contrast to many Western countries use of smokeless tobacco in women is quite common in rural areas of Pakistan. The primary objective of the present study was to determine the clinico-pathological features of histologically confirmed bladder carcinoma in women and the secondary objective was to investigate smokeless tobacco use as a possible risk factor for it.

## Patients and methods

Two hundreds and four patients of newly diagnosed bladder carcinoma were treated in the department of urology, Nishtar Hospital Multan, Pakistan from Jan 1998 to December 2004. Age, clinical presentation, cystoscopic findings and histopathological reports, and possible etiological risk factors of bladder carcinoma in women were studied prospectively.

After initial clinical evaluation and routine hematological, biochemical and radiological investigations all patients underwent cystoscopy and transurethral resection of bladder tumor (TURBT). All women were treated as inpatients and none of the patients had undergone TURBT previously. In all cases complete removal of papillary tumor was performed. In cases of solid muscle invasive tumors either complete resection or generous debulking of exophytic tumor was carried out. Cystoscopic tumor configuration was compared with the histopathological reports. Data were collected and prospectively updated at the time of discharge from hospital and during follow-up in urology out-patient clinic.

Detailed information about the smoking habits, use of smokeless tobacco (chewable or snuff), use of hair coloring dyes, occupational exposure to chemicals was obtained from all patient. Many of the women had long history of smokeless tobacco use, its duration and frequency was inquired from such patients. Possible effect of smokeless tobacco on the depth (T category) of bladder carcinoma in such patients was studied and compared with patients not using any form of tobacco.

## Results

Two hundred and four patients (160 male and 44 female with male female ratio 3.6:1) were treated. The age of the female patients ranged from 26–80 years (median age 55 years). Hematuria was the predominant symptom in 39 (88.6%) patients at the time of presentation. A total of 21 patients had painless and 18 patients had painful hematuria. Four patients presented with various urinary complaints but had no hematuria. In one patient the bladder tumor was incidentally detected on ultrasonography performed for some other complaints. The mean duration of symptoms was 4 months (range 2 weeks to 16 years)

At presentation mean hemoglobin concentration was 9.5 grams/dL (range 3.9 grams to 13.3 grams). Six (13.6%) patients had renal insufficiency (serum creatinine > 1.5 mg %) secondary to ureteric obstruction from bladder carcinoma. All patients had transitional cell carcinoma. Eleven (25%) patients had non-invasive superficial (i.e. pTa or pT1) transitional cell carcinoma while 33 (75%) patients had muscle invasive (T2–T4) transitional cell carcinoma. The median duration of symptoms for noninvasive transitional cell carcinoma was 1.5 years (range 2 weeks to 16 years) and it was 4 months (range 1 month to 2 years) for muscle invasive carcinoma.

Most superficial tumors had papillary and muscle invasive tumors had solid configuration at cystoscopy. Average size of the superficial and invasive tumor was 4 cm (range 1–8 cm) and 3.8 cm (range 2–8 cm) respectively. Of the superficial tumors 2 (18%) were pTa and 9 (82%) were pT1 tumors. Three pT1 tumors were grade I and five patients had grade II tumors. One patient had high-grade pT1 grade III carcinoma. There was no carcinoma *in situ *although no random biopsies were taken.

Of the muscle invasive tumors T2, T3 and T4 tumors were present in 14, 15 and 4 patients respectively. Thirteen patients with muscle invasive disease had histological grade III carcinoma while twenty patients had grade II carcinoma. None of the patients had GI tumor. Two patients had marked iliac and para-aortic lymphadenopathy while one patient had iliac and para-aortic lymphadenopathy and liver metastases at the time of diagnosis.

All females were non smokers but 21 (47%) patients had long history of smokeless tobacco use (moist snuff (*niswar*) 12 patients, chewable tobacco (*beera*) in 5 patients and chewed tobacco with betel nuts (*pan*) in 4 patients most of these were currently using these substances at the time of presentation. All such patients came from rural areas of Punjab and were uneducated. Patients were asked whether they were using smokeless tobacco 5 or less than 5 times/day or greater than 5 times /day.

By employing cross tabulation (table 1) of duration of smokeless tobacco use and depth (T category) of bladder carcinoma, it appears that majority of such patients had muscle invasive carcinoma at presentation and about 60% of patients have been using smokeless tobacco between 20–30 years. The correlation between the depth of bladder carcinoma and duration of smokeless tobacco use was 0.473 which is statistically significant (*p *= *0.03*). However correlation between bladder carcinoma and intensity of exposure to smokeless tobacco was 0.24 which showed a weak relationship. This might be due to the fact that the patients were using different quantities of smokeless tobacco from different sources.

**Table 1:** Depth (T category) of bladder carcinoma and duration of smokeless tobacco use

	**Duration of smokeless tobacco use**				
	**1–10 years**	**11–20 years**	**21–30 years**	**31–40 years**	**Total**

**T1 (pTa/pT1)**	1	2	1		4
**T2**		3	3		6
**T3**		2	6	1	9
**T4**			2		2
**Total**	1	7	12	1	21

We applied two independent sample *t *test for the comparison of depth of invasion. (by using T category of TNM stage) of bladder carcinoma in users and nonusers of smokeless tobacco. Patients using smokeless tobacco were assigned to group I and nonusers to group II (i.e. control group). The mean depth of bladder carcinoma in group I and II was 2.43 (standard deviation 0.93) and 2.13 (standard deviation 0.97) respectively. The datum shows that the carcinoma are of higher depth (T category) in group I, however the *t *value for the difference between the two groups is 1.04 which indicated that this difference was not statistically significant. Larger studies should be able to clarify the role of smokeless tobacco as an etiological risk-factor for bladder carcinoma.

## Discussion

Bladder carcinoma is the fourth most common cancer in men in the USA and eight most common cancers in women [4]. In Pakistan bladder carcinoma is one of the top ten malignancies in men and most common urological malignancy in both sexes [5]. Bladder cancer predominantly affects male, with a sex ratio of 3:1, suggesting sex-linked etiological factors [6]. In the present study the male female sex ratio was 3.6: 1. In women bladder cancer usually occurs above the age of 60 years [7], in the present study however the median age was 55 years and 68% women were less than 60 years of age.

A neoplastic change in the urothelium is a multi-step phenomenon [8]. The exact genetic events leading to this multi-step transformation are unknown, but they are likely to be multiple and may involve the activation of oncogenes and inactivation or loss of tumor suppression genes [9]

Cigarette smoking is the single most important cause of bladder carcinoma. Smokers have up to four fold higher incidence of bladder cancer than do people who never smoked [10]. The risk correlates with the number of cigarettes smoked, the duration of smoking and the degree of inhalation of smoke. Causative agents in cigarette smoke are thought to be alpha and beta naphthylamine, which are secreted in to urine of smokers [11]. When compared by number of cigarettes are smoked, the risk of bladder carcinoma may be higher in women than men [12] Cigarette smoking accounts for 50% and 31% of bladder cancers in men and women respectively [13]. Other forms of tobacco use are associated with only a slightly higher risk of bladder cancer [10].

In Pakistan 36% of men and 9% women are smokers [14]. Tobacco is also used in other forms such as hookah (hubble bubble), moist snuff used as an oral dip (niswar), chewed with betel nuts (pan) and smoking of rolled dry leaves containing tobacco (beedi). The most common form of tobacco use in women in rural Pakistan is chewing tobacco and snuff but because of cultural prohibitions women may under report use of tobacco [15]. In the present study none of the women were smokers but 47% women had history of intake of moist snuff (niswar) or chewable tobacco (beera and pan) and all came from the rural areas. The median duration of use of such tobacco products was 27 years (range 10–40 years).

Chewing tobacco and snuff contains many carcinogens. The most harmful carcinogens in smokeless tobacco are the tobacco specific nitrosamines (TSNA). They are formed during the growing, curing, fermenting and aging of tobacco [16]. Long term use of snuff can lead to a number of adverse health affects including oral cancer, cardiovascular diseases and gingival diseases [17]. However the etiological relationship between smokeless tobacco and bladder carcinoma has not been well elucidated in the literature and there is still no agreement among the researchers whether smokeless tobacco use enhances the risk of bladder cancer. Some studies reported increased risk of bladder cancer in smokeless tobacco users [18,19] while others [12] could find no such risk in smokeless tobacco users. All studies included smaller number of bladder cancer patients using smokeless tobacco.

In the present study 47% women had long history of smokeless tobacco use and the correlation between the stage of bladder carcinoma and duration of smokeless tobacco was significant (*p *= *0.03*). However, there was weak correlation between bladder carcinoma and intensity of exposure to smokeless tobacco. This might be due to the fact that women were using different quantities and forms of the locally available smokeless tobacco. As there is long latent period between exposure to carcinogens and the development of bladder carcinoma, it is possible that prolong use of smokeless tobacco among women in the present study was either the etiological factor or had modifying effect on its development. However further studies are required to clarify the role of smokeless tobacco in the development of bladder carcinoma.

In the USA percentage of bladder cancers attributed to occupational exposure is 21% for men and 11% for women [1]. None of the women in present study were exposed to occupational chemicals.

Majority of the patients with bladder carcinoma present with either hematuria or irritative voiding symptoms [20]. In the present study majority of women (88%) presented with hematuria while some (9 %) had variable urinary symptoms. In one patient the tumor was incidentally detected on ultrasonography.

In the developed world transitional cell carcinoma is reported for most bladder carcinoma. About 25% of newly diagnosed cancers are muscle invasive (T2–T4); the rest are superficial (70%), classified as limited to the mucosa (pTa), lamina propria (pT1) or being *in situ *(Tis 5%) [21].

In the present study all patients had transitional cell carcinoma. Some authors have reported that bladder cancer is of a higher stage at initial diagnosis in women [1]. In the present study 25% patients had superficial (pTa/pT1) and 75% patients had muscle invasive balder carcinoma. In a report from another Pakistani centre 97% of bladder carcinomas were muscle invasive [22] but clinico-pathological differences in women with bladder cancer were not separately reported. This high percentage of muscle invasive bladder carcinoma in Pakistan is in contrast to all other studies from USA and Europe.

Overall survival in patients with superficial disease is excellent. However 60%-70% of superficial tumors recur and 5% of pTa and 25% of pT1 tumors progress to invasive disease [21]. For recurrence risk, multiplicity of the tumor is the most important followed by recurrence rate, volume of the tumor, grade and T category. For progression the most important factor is the histological grade and the T category T1 GIII tumors carry poor prognosis and up to 50% progress to invasive disease [23].

Patients with tumors just invading detrusor muscles have a 50% five years survival whereas those whose tumors have invaded beyond the detrusor muscle have a 10% five years. At the time of diagnosis 50% of muscle invasive tumors have occult metastases which will manifest themselves clinically within 12 months; few such patients survive beyond two years [21]. In the present study two patients had marked iliac and para-aortic lymphadenopathy while one patient had iliac and para-aortic lymphadenopathy and liver metastases at the time of diagnosis.

## Conclusion

Transitional cell carcinoma is the most common bladder malignancy in women in Pakistan. Most women with bladder carcinoma have long history of use of smokeless tobacco. At the time of diagnosis 75% women have muscle invasive bladder carcinoma. In women using smokeless tobacco, there is significant correlation between stage of bladder carcinoma and duration of smokeless tobacco use. Further studies are required to clarify the role of smokeless tobacco in the development of bladder carcinoma.
